# Spectral Characteristics Simulation of Topological Micro-Nano Structures Based on Finite Difference Time Domain Method

**DOI:** 10.3390/nano11102622

**Published:** 2021-10-06

**Authors:** Xiaoran Ma, Bairui Du, Shengwang Tan, Haiying Song, Shibing Liu

**Affiliations:** 1 Strong-Field and Ultrafast Photonics Lab, Faculty of Materials and Manufacturing, Beijing University of Technology, Beijing 100124, China; maxiaoran@emails.bjut.edu.cn (X.M.); DUBR123@emails.bjut.edu.cn (B.D.); swtan@foxmail.com (S.T.); 2 Key Laboratory of Trans-Scale Laser Manufacturing Technology, Ministry of Education, Beijing University of Technology, Beijing 100124, China

**Keywords:** spectral characteristics, FDTD simulations, surface micro-nano structures

## Abstract

Natural structural colors inspire people to obtain the technology of spectral characteristics by designing and preparing micro-nano structures on the material’s surface. In this paper, the finite difference time domain (FDTD) method is used to simulate the spectral selectivity of micro-nano grating on an Au surface, and the spectral response characteristics of different physical parameters to the incident light are obtained. The results show that, when the grating depth is shallow, the absorption peaks of TM polarized incident light on the material surface take on redshifts with the increase in the grating period. Meanwhile, when the depth-width ratio of the grating structure is high, the absorption peak appears in the reflection spectrum and presents a linear red shift with the increase in the grating period after the linearly polarized light TE wave incident on the surface of the micro-nano structure. At the same time, the wavelength of the absorption peak of the reflection spectrum and the grating period take on one-to-one correspondence relations, and when the TM polarized light is incident, the reflection spectrum exhibits obvious selective absorption characteristic peaks at certain grating periods (for example, when the period is 0.4 μm, there are three absorption peaks at the wavelengths of 0.7, 0.95, and 1.55 μm). These simulation results can provide a good theoretical basis for the preparation of micro-nano structures with spectral regulation function in the practical application.

## 1. Introduction

Inspired by the structural colors of nature, it is found that, when the wavelength of the incident light wave is close to or smaller than the geometric size of the solid surface structure, peculiar optical phenomena often occur [1,2,3]. However, with the increasing demand for various special materials in many fields, the performance of natural materials has been unable to fully meet the needs of various special functions, and so scientific and technological workers have turned their attention to artificial processing methods. People have tried to achieve special performances for material surfaces by designing and fabricating periodic micro-nano structures on solid material surfaces [4,5,6,7,8].

In principle, when a light beam is incident on the surface of a material, due to the different performance parameters of the material surface, it is often accompanied by different degrees of absorption, refraction, and scattering, which leads to the redistribution of the surface light field. However, for the materials with periodic micro-nano structures on the surface, the incident light acting on the microstructure surface will cause the coupling of light wave energy, thus determining the final transmission direction and energy value of light wave. Therefore, the coupling effect can be controlled by adjusting the surface microstructure, and finally the effect of adjusting the photoelectric field can be achieved. The micro-nano structure generates colors by forming different geometric structures [9,10,11,12,13,14,15] and involves the tuning of resonance light in the visible wavelength range by manipulating the structural parameters [16,17,18,19]. Many subwavelength structural colors have been shown to have resolutions exceeding the diffraction limit [20,21,22,23]. In addition, through the preparation of micro-nano structures on the surface of the material, the absorption efficiency of light can be greatly improved, the reflection spectrum and absorption spectrum can be adjusted, and the spectral reflection or absorption at a specific optical frequency can be effectively controlled [24,25,26,27,28,29,30,31,32]. This method has been widely used in microelectronic systems [33,34], solar photovoltaic devices, military stealth, and other research fields [35,36,37,38]. 

A topological characteristic structure refers to a structure with three-dimensional spatial geometric symmetry in a semi-infinite space. The supernormal optical (electromagnetic) properties of the surface with subwave-length topological characteristic structure are essential to realize the special surface performance. As a topological characteristic structure, the micro-nano grating structure is also a typical representative of a periodic micro-nano structure. Under certain conditions, there will be surface phonon resonance, surface plasmon resonance enhancement, a dielectric confinement effect, etc. These effects will enhance the absorption of light. Therefore, this study is mainly based on the finite difference time domain method (FDTD) to perform spectral simulation calculations on the micro-nano grating structure on the surface of metallic gold, and simulate the influence of various physical characteristic parameters of the structure on the surface spectrum, to explore more periodic topological characteristic structures with spectral characteristics and provide a forerunner for practical engineering applications. 

## 2. Simulation Method and Model Design

FDTD is currently one of the most commonly used numerical calculation methods for studying the interaction process of light and complex structures. In contrast to the traditional analytical solution with more analytic approximation, the FDTD method has high computational accuracy with a simple algorithm and the limited applicability of analytical approximation models can be avoided, so it has a wide range of applications in the field of computational electromagnetism. The FDTD Solutions used in here is based on FDTD to solve Maxwell’s equations. It can be used in the design, analysis, and optimization of micro-nano photonics such as micro-nano photonic devices and micro-nano optical materials. In terms of material selection, compared with other metallic materials such as Fe, Al, Ag, etc., gold (Au), a precious metal, has unique properties related to light, electricity, and catalysis, and has application potential in the fields of new energy, optoelectronics, and biomedicine. Among these applications, more attention has been paid to the research and application exploration of its optical properties, such as spectroscopy, photovoltaic effect, photodetection, and biosensing. Therefore, in the simulation experiment in this article, metallic gold is selected as the material to study the selective absorption or reflection influence of its surface micro-nano characteristic structure on the reflection spectrum. 

**Figure 1:**
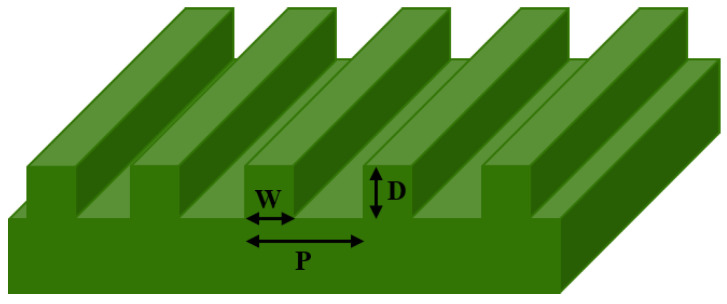
The model of the Au grating structure.

The micro-nano grating structure modeling used for simulation is shown in Figure 1. The grating structure characteristics are the grating period, grating width, and the grating depth, and the incident light source is a plane wave with wavelength λ. Since the grating structure is a periodic structure, the FDTD simulation process is carried out in a two-dimensional plane. The simulation range is a single period. The x-axis and y-axis directions are set to periodic boundary conditions and perfectly match layer (PML) ideal boundary conditions, respectively, and the simulation accuracy is *λ*/12. For the interface between the metallic material and the air, a mesh grid is added for local refinement analysis, the grid accuracy is set to 10 nm, and a suitable field power monitor is selected to monitor and analyze the reflection spectrum of the sample surface. In the simulation process, optical simulation was performed with different microstructure parameters, such as grating period (P), width (W), depth (D), duty ratio (W/P), light source wavelength (λ) and light source polarization angle (A).

**Figure 2:**
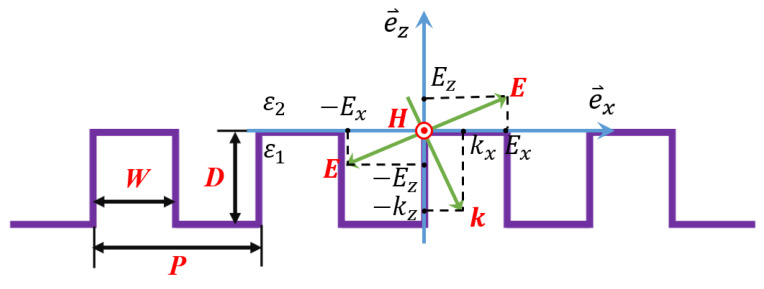
Schematic diagram of interaction between light (electromagnetic wave) and surface micro-nano grating.

As it is known that the surface state and bulk state of the material have different optical properties, according to the point group theory, the symmetry breaking of surface states will result in a significant nonlinear optical effect of the material surface, meaning that the interaction between light and the material surface will become more complex. However, the existence of surface residual bonds and the asymmetry of atomic structure cause the tension of the linear chain to change, and cause a change in lattice vibration (oscillator) frequency. There are abundant free electrons on the metal’s surface, under external disturbances (light and thermal effects, etc.). Figure 2 shows a schematic diagram of the interaction between light and surface micro-nano grating; on the one hand, these free electrons can easily cause density fluctuations on the surface, and the charge density fluctuations in the longitudinal direction (z direction) are shielded in the Thomas–Fermi length of about 1 Å, which is called surface plasmon oscillation. On the other hand, this charge density fluctuation outside the surface (|z|>0) will be accompanied by a transverse electromagnetic field [39,40]. In this paper, the micro-nano grating on the surface of metallic gold can be equivalent to the plasma with uneven distribution on the surface; that is, the density gradient ($\vec{\nabla }n_{e}$) is generated. The grating period is the characteristic scale *P* of the plasma. If the incident light excites the electron oscillation, the light wave electric field is needed to generate a component in the direction of the electron density gradient, and a component of the electric vector can cause the electrons to oscillate in the direction of the density gradient—that is, $\vec{E}\cdot \vec{\nabla }n_{e}\neq 0$. This oscillation generates the charge density fluctuation, which can be strengthened by the plasma resonance and generate resonant absorption. When the transverse electric (TE) wave (electric field perpendicular to the plane of incidence) is incident we find that the reflected light wave corresponds to the grating period when the grating depth is shallow, which indicates that resonance occurs between the incident light wave and the lattice vibration of the surface with the same frequency at this time. Therefore, the wavelength of the reflected wave is the same as the equivalent characteristic length of the surface plasma. When the incident light source is a transverse magnetic (TM) wave, the existence of the grating structure will introduce an additional wave vector to meet the wave vector matching conditions, and then realize the selective absorption and reflection of light waves at different wavelengths (Appendix A). 

## 3. Results and Discussion

**Figure 3:**
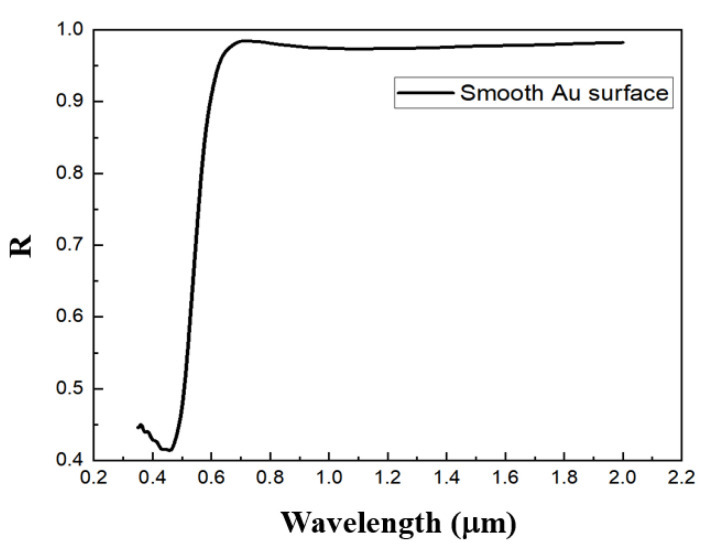
Reflection spectrum simulation result of the smooth Au surface.

The refractive index parameters of the precious metal gold used in the simulation are obtained from the database CRC of the software. Figure 3 shows the simulation results of the reflection spectrum of the smooth Au surface without a structure, from which we can see that there is hardly any absorption. 

### 3.1. The Influence of Grating Depth–Width Ratio on Reflection Spectra

**Figure 4:**
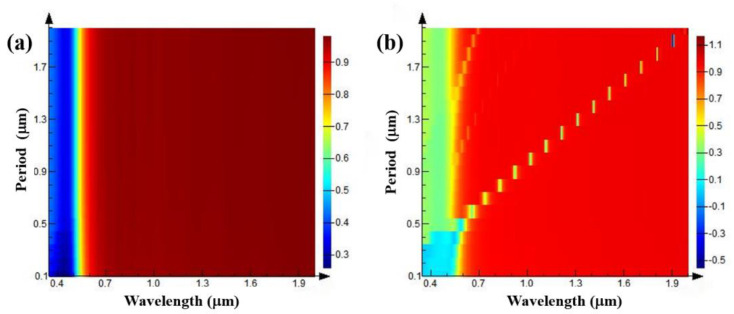
Reflection spectra of gratings with different periods when the grating depths are 0.5 μm, the grating duty ratios are 0.5, and TE (**a**) and TM (**b**) polarized light are incident.

First, the periods of the grating structure are set to 0.1–2 μm, the grating depth to 0.5 μm, the wavelength of the light source to 0.4–2 μm, the duty ratio of the grating structure is kept at 0.5, and the reflection spectrum is simulated and analyzed. As shown in Figure 4a, compared with the smooth Au surface (Figure 3), when the polarization direction of the incident light source is TE polarization, in the wavelength range of 0.4–2 μm, the reflection spectrum hardly changes. However, when the polarization direction of the incident light source is TM polarization, it can be seen from Figure 4b that a strong absorption peak appears when the grating period is 0.5 μm. With the gradual increase in the grating period, the absorption peak of the reflection spectrum exhibits a linear red shift, and the linewidth of the absorption peak gradually narrows and the absorption intensity gradually increases. When the grating period reaches about 1.3 μm, a second-order absorption peak appears. The intensity of the absorption peak is relatively weak and a linear red shift also appears with the increase in the grating period. 

**Figure 5:**
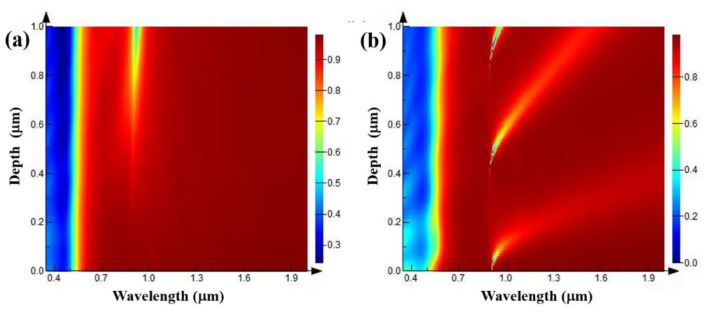
Reflection spectra of gratings with different depths when the grating periods are 1 μm, the grating duty ratios are 0.5, and TE (**a**) and TM (**b**) polarized light are incident.

Select the grating with a period of 1 μm and a width of 0.5 μm to study the variation influence of the grating depth for its spectral characteristic. At this time, the grating duty ratio is 0.5, the wavelength of the light source is the same as above. The grating depth is set to continuously change within the range of 0.05–1 μm. The simulation results of the reflection spectra are shown in Figure 5. Figure 5a shows the variation of the reflection spectrum with the grating depth when the TE polarized light is incident. It can be seen that the reflection spectrum does not change when the grating depth is less than 0.6 μm. When the grating depth is greater than 0.7 μm, with the increase in grating depth, the reflection spectrum appears an absorption peak at 0.7 μm, and the absorption peak gradually increases with the increase in grating depth. Figure 5b shows the variation of the reflection spectrum with the grating depth when the TM polarized light is incident. It can be seen from the figure that when the grating depths are 0.05, 0.5, and 0.9 μm, respectively, there are reflection spectrum absorption peaks, which are called the first-order, second-order, and third-order absorption peaks, respectively. At the same time, in a certain range of grating depth, the absorption peak of the reflection spectrum gradually redshifts with the increase in grating depth, and the peak intensity of the absorption peak decreases, while the FWHM (full width at half maximum) of the absorption peak increases. 

**Figure 6:**
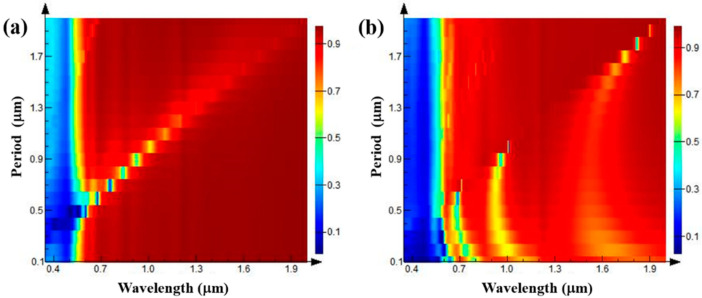
Reflection spectra of gratings with different periods when the grating depths are 1 μm, the grating duty ratios are 0.5, and TE (**a**) and TM (**b**) polarized light are incident.

An absorption peak appears in the reflection spectrum with the increase in grating depth when TE polarized light is incident and the grating depth is greater than 0.7 μm. Therefore, further optical simulation of the phenomenon in the situation with the deeper grating was performed. The grating depth is set to 1 μm, the grating duty ratio is kept at 0.5, the grating period is set to 0.1–2 μm, and the incident light is still set to TE polarization light and TM polarization light. The simulation results are shown in Figure 6.

**Figure 7:**
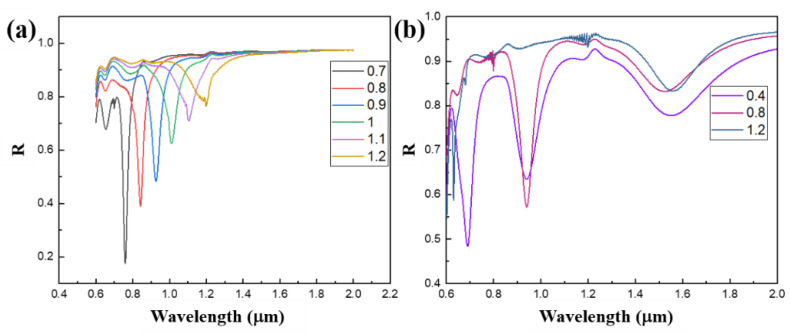
Reflection spectra curves of gratings with different periods drawn from partial data extracted from Figure 6, (**a**) and (**b**) are TE and TM polarized light incident respectively.

It can be seen from Figure 6a that, when the incident light is TE polarization, a strong absorption peak appears at the grating period of 0.7 μm. In the 0.5–2 μm wave band, the absorption peak begins to move toward the long band with the gradual increase in the grating period, the linewidth of the absorption peak increases gradually, and the intensity decreases gradually. When the grating period reaches 1.4 μm, the absorption peak is very weak, until it disappears. When the TM polarized light is incident, as shown in Figure 6b, strong absorption peaks appear at the grating periods of 0.7, 0.95, and 1.7 μm, corresponding to the light wavelengths of 0.7, 0.95, and 1.75 μm, respectively. By comparing these with the incident situation of TE polarized light, it can be clearly seen that with the increase in grating period, the peak points of the absorption peaks of the reflected light are discretely distributed in the same linear relationship as the results obtained when TE polarized light is incident. In order to more clearly see the change in absorption peak intensity, some of the data in Figure 6 are extracted and plotted in the graph shown in Figure 7.

**Figure 8:**
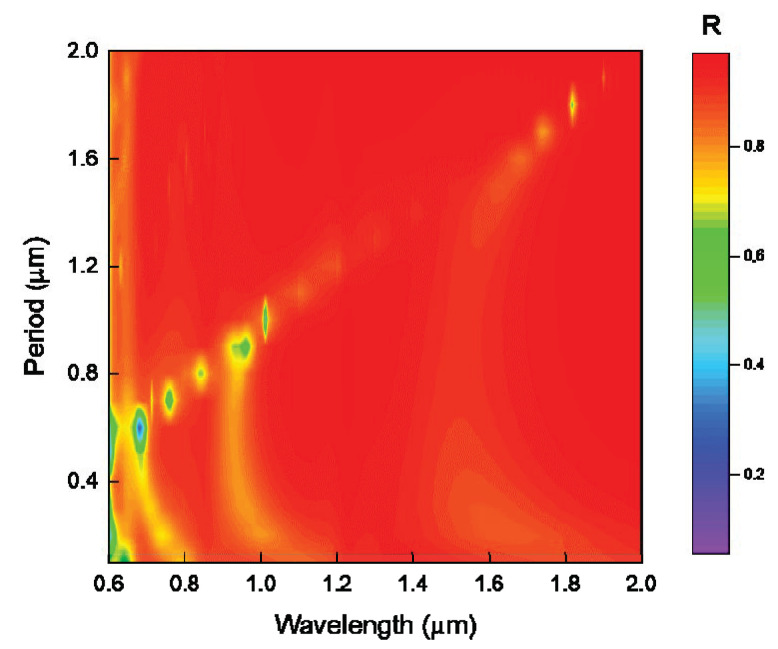
Reflection spectra of gratings with different periods when the grating depths are 1 μm, the grating duty ratios are 0.5, and natural light is incident.

Six reflection spectra with grating periods of 0.7, 0.8, 0.9, 1, 1.1, and 1.2 μm are selected, as shown in Figure 7a, at wavelengths of 0.75, 0.85, 0.95, 1, 1.1, and 1.2 μm corresponding to the absorption peaks, respectively. As shown in Figure 7b, three reflection spectra with grating periods of 0.4, 0.8, and 1.2 μm are selected in the graph. It can be seen that there are three typical absorption peaks near the wavelengths of 0.7, 0.95, and 1.55 μm. When the grating period is 0.4 μm, the absorption peaks with grating periods of 0.8 and 1.2 μm are included. Similarly, when the grating period is 0.8 μm, only the absorption peak with a grating period of 1.2 μm is included. In other words, at discrete wavelengths, the small-period grating structure can contain the absorption mode of the large-period grating structure—that is, when the wavelength is the same, at the linear coupling point between the grating period and the wavelength, the absorption peak of the reflected light remains basically unchanged with the decrease in the grating period. In addition, according to the simulation characteristics of FDTD, in the simulation process, both the TE polarized incident light source and the TM polarized incident light source are applied at the same time, and the data obtained are the data after the two simulation results in Figure 6 are superimposed. That is, the chart of the reflection spectrum changing with the period when natural light is incident is obtained, as shown in Figure 8; it can be seen that the absorption peak of the reflection spectrum has a linear red shift with the increase in period, and the wavelength of the absorption peak has obvious selectivity for the grating period. For example, when the grating period increases to 0.8 μm, the absorption peak at 0.7 μm wavelength disappears.

**Figure 9:**
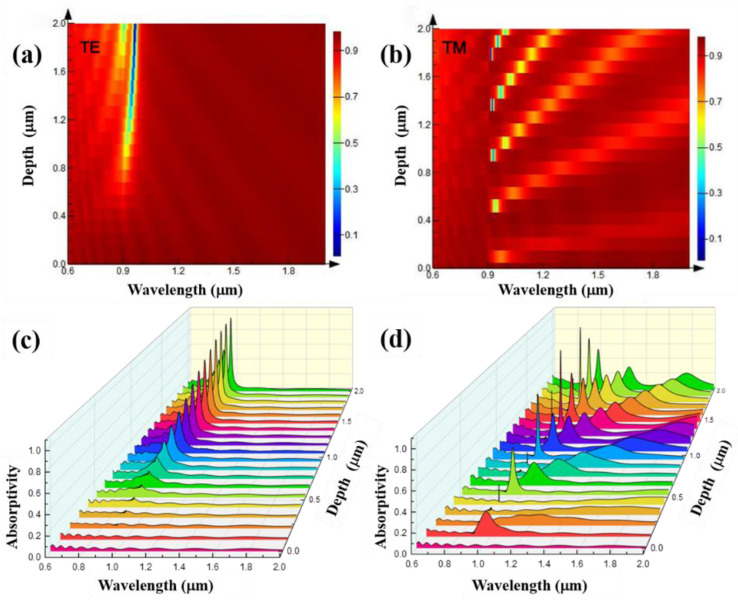
When the grating periods are 0.9 μm, the grating duty ratios are 0.5, and the reflection spectra of gratings with different depths when TE (**a**) and TM (**b**) polarized light are incident; absorption spectra of gratings with different depths when TE (**c**) and TM (**d**) polarized light are incident.

Based on the results of Figure 5 and Figure 6, the influence of grating depth changes was further explored. The grating period is set to 0.9 μm, the grating duty ratio remains 0.5, and the grating depth is set to 0.05 μm–2 μm, changing continuously, and the simulation result is shown in Figure 9. When the TE polarized light is incident and the grating depth is less than 0.8 μm, the reflection spectrum of the grating structure is almost unchanged with the change in the grating depth. When the grating depth is above 0.8 μm, as shown in Figure 9a,c, it is found that an absorption peak appears at the wavelength of 0.9 μm, and the absorption peak becomes narrower and stronger with the increase in the grating depth. When the TM polarized light is incident, as shown in Figure 9b,d, as the grating depth increases, high-order discrete absorption peaks such as of the second and third order appear in sequence, and the discrete absorption peaks redshift as the grating depth increases. At the same time, the intensity of the absorption peak decreases gradually and the linewidth increases gradually.

### 3.2. The Influence of Grating Duty Ratio on Reflection Spectra

**Figure 10:**
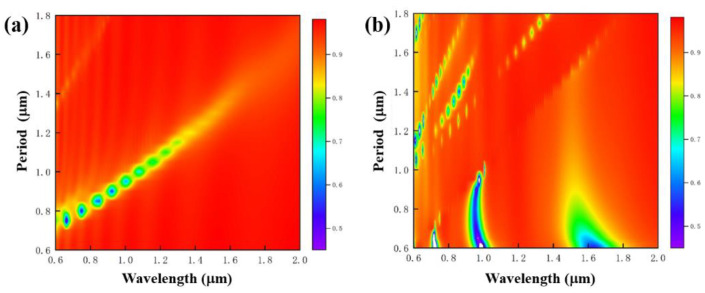
Reflection spectra of high depth–width ratio gratings with different periods when the grating depths are 1 μm, the grating widths are 0.45 μm, and TE (**a**) and TM (**b**) polarized light are incident.

First, when the depth–width ratio of the grating is relatively high, the influence of different grating periods—that is, the duty ratio—on the reflection spectra is explored. The grating width remains 0.45 μm, the grating depth is 1 μm, and the grating period changes from 0.6 to 1.8 μm—that is, the duty ratio changes from 0.75 to 0.25. According to the simulation results shown in Figure 10a, when the incident light is TE polarization, there will be an absorption peak of reflected light at the wavelength corresponding to the grating period. With the increase in the grating period, the absorption peak of the reflection spectrum also shows a linear red shift. At the same time, the peak intensity of the grating absorption peak gradually decreases, and the FWHM gradually increases. When the incident light is TM polarization, as shown in Figure 10b, the linear red shift of some absorption peaks can still be found, but the other part of the absorption peak does not change with the increase in grating period, and the central wavelength of the absorption peak of the reflected light is discretely distributed with the same linear relationship as the result obtained when the TE polarized light is incident. 

### 3.3. The Influence of Light Source Polarization Angle on Reflection Spectra

**Figure 11:**
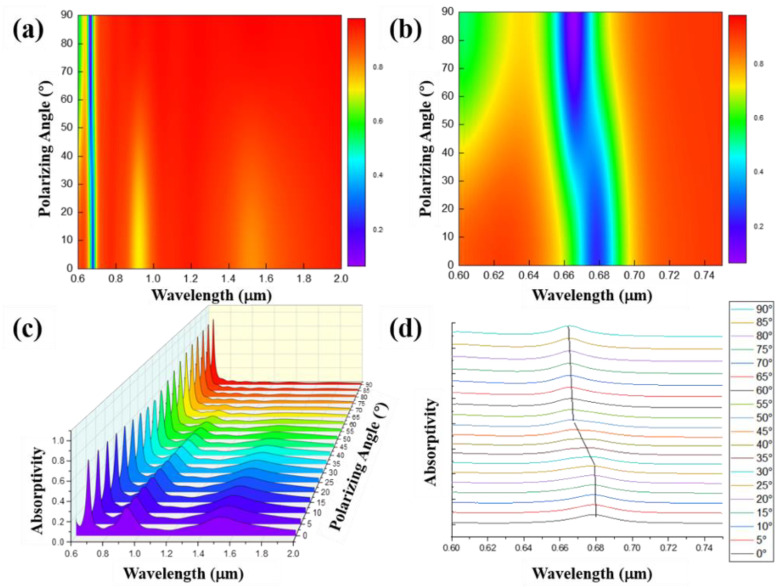
Reflection spectrum (**a**) and absorption spectrum (**c**) of high depth–width ratio gratings when the grating depths are 1 μm, the grating widths are 0.3 μm, the grating periods are 0.6 μm, the grating duty ratios are 0.5, and plane waves with different polarization angles are incident; (**b**) is the partial image of (**a**); (**d**) is the two-dimensional image of (**c**).

Next, still maintaining a high depth–width ratio, the reflection spectra of a grating structure with a grating width of 0.3 μm, a period of 0.6 μm, a depth of 1 μm, and a duty ratio of 0.5 under different polarized light incidence conditions were simulated. The light source is set as linearly polarized light, and the polarization angle is set to 0–90° (i.e., from the TM polarization direction to the TE polarization direction) with 5° as the interval for simulation. The results are shown in Figure 11. It can be seen from the figure that there are two strongest absorption peaks at the polarization angles of 0° and 90°. With the change in the polarization direction of the electrical field, the absorption peak of TM polarized light at 0° gradually weakens until the polarization angle is around 45°. At this time, the absorption peak of TE polarized light appears and continuously increases to the maximum when the polarization angle is 90°.

**Figure 12:**
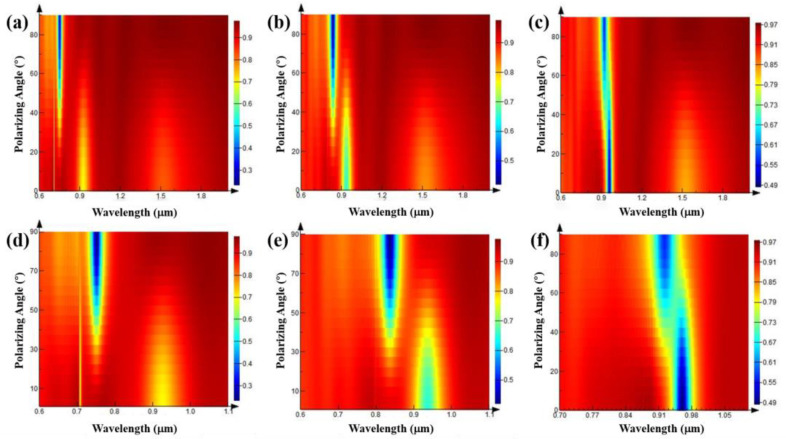
Reflection spectra of high depth–width ratio gratings with different periods when the grating depths are 1 μm, the grating duty ratios are 0.5, and plane waves with different polarization angles are incident. (**a**) Grating width is 0.35 μm, period is 0.7 μm; (**b**) grating width is 0.4 μm, period is 0.8 μm; (**c**) grating width is 0.45 μm, period is 0.9 μm; (**d**–**f**) are the partial images of (**a**–**c**), respectively.

As the central wavelength of TE polarization absorption peak is close to that of TM polarization absorption peak when the grating width is 0.3 μm and the grating period is 0.6 μm, we tried the microstructures when the grating periods are 0.7, 0.8, and 0.9 μm, respectively. Set the grating width to 0.35, 0.4, and 0.45 μm to keep the duty ratios consistent; both are 0.5, and the grating depths are 1 μm. The light source is set as linearly polarized light, and the polarization angle is set to 0–90° with 5° as the interval for simulation. The results are shown in Figure 12. In these three cases, there are two strong absorption peaks at the two polarization angles of 0° and 90°. As the polarization direction of the electric field changes, the absorption peak of TM polarized light at 0° gradually weakens until the polarization angle is around 45°. At this time, the TE polarized light absorption peak appears and continues to increase, reaching the maximum when the polarization angle is 90°. The conclusion obtained here is consistent with the conclusion when the grating period is 0.6 μm. 

## 4. Conclusions

The FDTD is an important method foundation for engineering application to design and fabricate the characteristic structure on a material’s surface required by the expected optical properties (such as negative dielectric constant or negative permeability, etc.). In this paper, the FDTD method is used to simulate the change in the reflection spectrum characteristics and the selective spectrum length caused by different geometric characteristic parameters when the micro-grating structure is directly constructed on the metallic Au surface. The results show that when the grating depth is shallow and the incident light source is TE polarized light, the reflection spectrum does not change with the increase in the grating period. When the incident light source is TM polarized light, the absorption peak redshifts with the increase in the grating period with the same proportion. However, when the depth–width ratio of the grating structure is high, the abnormal absorption peak appears in the reflection spectrum after the linearly polarized light TE is incident on the structured surface, and with the increase in grating period, the absorption peak linearly shifts to red, the wavelength of the absorption peak of the reflection spectrum has a close and one-to-one correspondence with the grating period; when the TM polarized light is incident, the reflection spectrum exhibits obvious selective absorption characteristic peaks at certain grating periods (for example, when the period is 0.4 μm, there are three absorption peaks at the wavelengths of 0.7, 0.95, and 1.55 μm). In particular, the wavelength of the absorption peak has obvious selectivity for the grating period. For example, when the grating period is increased to 0.8 μm, the absorption peak at the wavelength of 0.7 μm disappears. These simulation results can provide a good theoretical basis for us to directly prepare the surface micro-nano structure of the material in the later stage.

## Data Availability

Data can be available upon request from the authors.

## Appendix A

Here we use Maxwell’s equations to further analyze the interaction process between light waves and surface plasma and the dispersion relationship [38,39]. The differential form of Maxwell’s equations is:

(A1) $$\{\begin{array}{c} \nabla \times \vec{H}=\sigma \vec{E}+\varepsilon_{0}\varepsilon \frac{\partial \vec{E}}{\partial t}\\ \nabla \times \vec{E}=-\mu_{0}\frac{\partial \vec{H}}{\partial t}\\ \nabla \cdot \vec{H}=0\\ \nabla \cdot \vec{E}=\rho \end{array},$$

where *H* is the magnetic field intensity, *E* is the electric field intensity, *σ* is the conductivity of the medium, *ε* is the dielectric constant of the medium, and ρ is the bulk charge density of the medium. Take the curl of the first two terms in Maxwell’s equations, we can get the following equation:

(A2) $$\{\begin{array}{c} \nabla^{2}\vec{E}-\mu_{0}\varepsilon \varepsilon_{0}\frac{\partial^{2}\vec{E}}{\partial t^{2}}=0\\ \nabla^{2}\vec{H}-\mu_{0}\varepsilon \varepsilon_{0}\frac{\partial^{2}\vec{H}}{\partial t^{2}}=0 \end{array},$$

Let the electric field be $\vec{E}=\left(E_{x}+E_{y}+E_{z}\right)e^{-i\omega t}$ and the magnetic field be $\vec{H}=\left(H_{x}+H_{y}+H_{z}\right)e^{-i\omega t}$. Two sets of independent solutions of Equations (A3) and (A4) can be obtained by simplifying the equation. The solution of TM wave is expressed by $H_{y}$, $E_{z}$, $E_{x}$, and the solution of TE wave is expressed by $E_{y}$, $H_{x}$, $H_{z}$:

(A3) $$\{\begin{array}{c} E_{x}=-i\frac{1}{\omega \varepsilon \varepsilon_{0}}\frac{\partial H_{y}}{\partial z}\\ E_{z}=-i\frac{\beta }{\omega \varepsilon \varepsilon_{0}}H_{y}\\ \frac{\partial^{2}H_{y}}{\partial z^{2}}+\left(k_{0}^{2}\varepsilon -\beta^{2}\right)H_{y}=0 \end{array},$$

(A4) $$\{\begin{array}{c} H_{x}=i\frac{1}{\omega \mu }\frac{\partial E_{y}}{\partial z}\\ H_{z}=\frac{\beta }{\omega \mu }E_{y}\\ \frac{\partial^{2}E_{y}}{\partial z^{2}}+\left(k_{0}^{2}\varepsilon -\beta^{2}\right)E_{y}=0 \end{array},$$

For the TM wave solution equation, substitute reasonable boundary conditions: $H_{y_{1}}=H_{y_{2}},\;E_{x_{1}}=E_{x_{2}}$. When  z>0, set the wave vector of the electric field is $k_{1}$, and the dielectric constant is $\varepsilon_{1}$. When z<0, set the wave vector of the electric field is $k_{2}$ and the dielectric constant is $\varepsilon_{2}$.

Therefore, the boundary conditions are:

(A5) $$\frac{k_{2}}{k_{1}}=-\frac{\varepsilon_{2}}{\varepsilon_{1}},$$

By substituting the boundary conditions into the Equation (A3), the dispersion relation of plasma wave when TM wave is incident can be obtained as follows:

(A6) $$k_{SPP}=\beta =k_{0}\sqrt{\frac{\varepsilon_{1}\varepsilon_{2}}{\varepsilon_{1}+\varepsilon_{2}}},$$

Similarly, the dispersion relation of plasma wave when TE wave is incident can be obtained:

(A7) $$E_{y}\left(k_{z_{1}}+k_{z_{2}}\right)=0,$$

Assuming that TE wave exists in the form of surface wave, $k_{z_{1}}$ and $k_{z_{2}}$ are both positive numbers. At this time, only when $E_{y_{1}}=E_{y_{2}}=0$ satisfies the assumption conditions, the surface plasma cannot be excited when the perpendicular incident light source is TE wave. Therefore, the surface electrons or plasmas are more easily excited by TM wave.
